# Semantic Annotation for Biological Information Retrieval System

**DOI:** 10.1155/2015/597170

**Published:** 2015-02-09

**Authors:** Mohamed Marouf Z. Oshaiba, Enas M. F. El Houby, Akram Salah

**Affiliations:** ^1^Computer Science Department, Faculty of Computers and Information, Cairo University, Dr. Ahmed Zewail Street, Orman, Giza 12613, Egypt; ^2^Engineering Division, Systems & Information Department, National Research Centre, El Buhouth Street, Dokki, Cairo 12311, Egypt

## Abstract

Online literatures are increasing in a tremendous rate. Biological domain is one of the fast growing domains. Biological researchers face a problem finding what they are searching for effectively and efficiently. The aim of this research is to find documents that contain any combination of biological process and/or molecular function and/or cellular component. This research proposes a framework that helps researchers to retrieve meaningful documents related to their asserted terms based on gene ontology (GO). The system utilizes GO by semantically decomposing it into three subontologies (cellular component, biological process, and molecular function). Researcher has the flexibility to choose searching terms from any combination of the three subontologies. Document annotation is taking a place in this research to create an index of biological terms in documents to speed the searching process. Query expansion is used to infer semantically related terms to asserted terms. It increases the search meaningful results using the term synonyms and term relationships. The system uses a ranking method to order the retrieved documents based on the ranking weights. The proposed system achieves researchers' needs to find documents that fit the asserted terms semantically.

## 1. Introduction

The emergence of information and communication technologies has drastically changed biological scientific research processes. As a consequence, the variety of biological data available in the public domain is now very diverse and ranges from genomic-level high-throughput data to molecular-imaging studies to published research articles. The paradox of such an expansion is that biological researchers now face the problem of extracting the specific data they need [1]. Furthermore, biological publications hardly follow naming conventions for entities and remain attached to their author's favorite names [2].

The Semantic Web technology changes the way search engines work. The traditional way used by search engines to find documents is by using the syntax or the key phrase of the word. So when the search engine adds some intelligence by using the semantic search it will improve the search results. Semantic technology uses the word in addition to the meaning of the word, relations with other words, word roots, and other linguistic synonyms to be understood by the computers or people. Metadata is a powerful base used in the Semantic Web. Each document can contain some related words that help to find it easily in the search process [3].

The semantic technology relies heavily on the formal ontologies that structure underlying data for the purpose of comprehensive and transportable machine understanding. Therefore, the success of the semantic technology depends strongly on the proliferation of ontologies, which requires fast and easy engineering of ontologies and avoidance of a knowledge acquisition bottleneck. Conceptual structures that define an underlying ontology are germane to the idea of machine processable data on the Semantic Web [4].

Ontology is specification of conceptualization for specific domain used in knowledge sharing purposes. They are (meta)data schemas, providing a controlled vocabulary of concepts, each with explicitly defined and machine processable semantics. By defining shared and common domain theories, ontologies help both people and machines to communicate concisely, supporting the exchange of semantics and not only syntax [4].

Biological researchers have spent a lot of time in building ontologies because of their importance in the field of information retrieval. They used ontologies and terminologies to describe their data and turn it into structured and formalized knowledge. Ontology is used as unified protocol to share the knowledge between different resources. One of the most familiar biological ontologies is the gene ontology (GO). It provides ontology of defined terms representing gene product properties. It covers three domains: cellular component (CC), the parts of a cell or its extracellular environment; molecular function (MF), the elemental activities of a gene product at the molecular level, such as binding or catalysis; and biological process (BP), operations or sets of molecular events with a defined beginning and end, pertinent to the functioning of integrated living units: cells, tissues, organs, and organisms as provided in the gene ontology [5].

Document annotation aims at discovering documents in references automatically. It is quite useful for many tasks including information extraction, classification, text summarization, question answering, and literature-based knowledge discovery. On the other hand, the web as a global information space is developing from a web of documents to a web of data. Currently, there are billions of publicly available web data sources of different domains. Biological domain is one of the rich data domains. Most available biological data are in unstructured format and described without any ontology concepts. These data sources are becoming more tightly interrelated as the number of links in the form of mappings grows [6].

The main objective of this research is to use the semantic technology in biological field to improve search results. The proposed system's purpose is to find documents that contain molecular function or biological process that can be applied on specific cellular component. The proposed system helps biological researchers to find relevant documents to their asserted terms in high performance. To fulfill this objective GO has been decomposed into three subontologies (CC, MF, and BP), which enable the researchers to select the searched terms from three different subontologies and give them more flexibility to select any combination of the three subontologies terms.

An annotation index has been created to speed the searching process, which annotates each document based on gene ontology biological concepts. In this research annotation refers to describe each document with gene ontology concepts and synonyms.

The query expansion has been used to increase the proposed systems' intelligence. The inferred terms from ontology are used to expand query to give an advantage in reaching more accurate results by retrieving documents that contain inferred terms related to asserted terms.

This research consists of the following. (I) Divide the gene ontology semantically to its three subontologies (molecular functions, cellular component, and biological processes). (II) Annotate the biological documents using the subontologies concepts and build an annotated index to refer to documents' terms and accelerate the time of searching. (III) Search for the needed documents in the annotated index using any combination of the selected subontology terms or any individual subontology term.

The remainder of this paper is organized as follows: Section 2 overviews some of related work.  Section 3 discusses the proposed system description and framework levels.  Section 4 is an illustrative example of the framework showing inputs and outputs of the proposed system.  Section 5 conducts experimental results on the proposed system.  Section 6 is the conclusion and future work.

## 2. Related Work

A lot of scientific researches have tackled the semantic technology and its relation with the information retrieval field. There are several tools that support the retrieval of information based on biological content. In [2] they Introduced GeneView, which built upon a comprehensively annotated version of PubMed abstracts and openly available PubMed Central full texts. The semistructured representation of biological texts enables a number of features extending classical search engines. For instance, users may search for entities using unique database identifiers. Annotation is performed by a multitude of state-of-the-art text-mining tools for recognizing mentions from 10 entity classes and for identifying protein-protein interactions. GeneView currently contains annotations for >194 million entities from 10 classes for ~21 million citations with 271000 full text bodies.

Another tool used to annotate and index biological documents is described in [1]. The key functionality of this system is to provide a service that enables users to locate biological data resources related to particular ontology concepts. The system's indexing workflow processes the text metadata of diverse resource elements such as gene expression data sets, descriptions of radiology images, clinical-trial reports, and PubMed article abstracts to annotate and index them with concepts from appropriate ontologies. The system enables researchers to search biological data sources using ontology concepts.

Using the web crawler to annotate data is also an important method in annotation and extraction. This technique is shown in SOBA (SmartWeb Ontology-Based Annotation) [7] which is a component for ontology-based information extraction from soccer web pages for automatic population of a knowledge base that can be used for domain-specific question answering. SOBA realizes a tight connection between the ontology, knowledge base, and the information extraction component. The originality of SOBA is in the fact that it extracts information from heterogeneous sources such as tabular structures, text, and image captions in a semantically integrated way. In particular, it stores extracted information in a knowledge base and in turn uses the knowledge base to interpret and link newly extracted information with respect to already existing entities.

In [8], they focus on the query expansion instead of document ranking. The model is parsing each topic into the event part and the geographic part and use different ontologies to expand both parts, respectively.

In [9], the researchers presented a framework for a semantic biological retrieval system using inverted list that effectively searches and retrieves meaningful results based on gene ontology. The framework takes two biological terms as input and retrieves the relevant documents as output. It improves the search result by expanding the biological terms using inferred terms (synonyms, parent, and grandparent) to retrieve more meaningful results. The framework uses a special ranking methodology to give weight to each document based on the rank value to help in ordering the result.

Many other researchers used ontologies, annotation, and information retrieval system using different techniques [3, 6, 10–14].

The previous work is concentrated on the ontologies that try to annotate the literature papers, optimize the searching process, increase the search result using the query expansion method, and increase the recall and precision of the retrieved document. Our proposed system is a semantic biological information retrieval system that annotates biological documents using multiontology. It is mainly directed to researchers that need to find documents that contain semantic relationships among the three main concepts in GO (cellular component, biological process, and molecular function). The system uses the term's relations and term's synonyms to give more meaning results. Ranking methodology is used in this system to help the researchers to find the most appropriate result first. It is our assumption that the system will help the researcher by minimizing the time and effort of searching semantically for any combination of the three subontologies concepts.

After reviewing several scientific researches that concentrate on the semantic technology, query expansion, gene ontology, and annotation, we conclude that the closer researches to this research are [1, 9]. Reference [1] is close to this research from the annotation perspective and semantic perspective. It presents a system for ontology-based annotation and indexing of biomedical data. It uses ontology to automatically expand the initial set of annotations generated by a concept recognition tool. Reference [9] is close to this research from the semantic perspective. Their framework is a semantic biological retrieval system that depends on gene ontology. It uses terms' synonym, parent, and grandparent to semantically enhance the system results using query expansion method.

## 3. Proposed System

**Figure 1:**
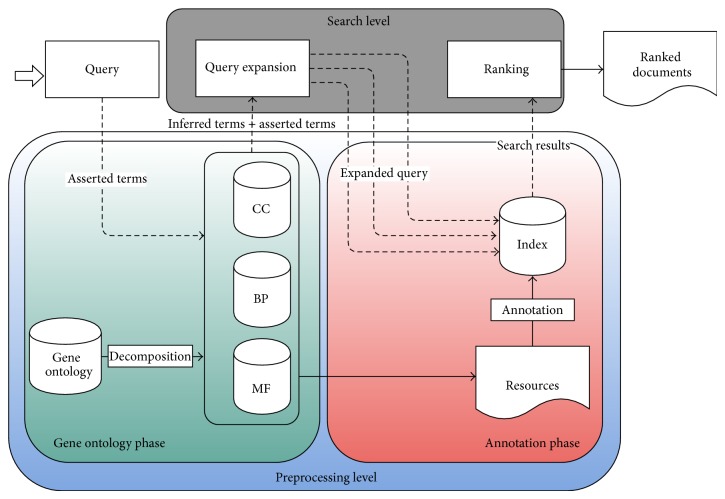
The framework of the proposed system.

The proposed system is an information retrieval system that searches for biological documents relevant to the asserted terms in the annotated index. Since the main purpose of this research is to retrieve documents that contain function and/or process for specific cellular component, it decomposes the gene ontology to its three subontologies and uses them to annotate the biological documents and builds the annotated index. The annotated index can be used as the metadata of each document. Semantics has been used in the proposed system to enhance the search results by adding terms' synonyms and relations from the gene ontology to the query to gain more semantic results. After searching the index and getting the results, the system ranks the results with specific criteria. The proposed system contains two levels which are preprocessing level and search level as shown in Figure 1.

### 3.1. Preprocessing Level

Preprocessing is the first level in the proposed framework. It consists of two phases which are gene ontology phase and annotation phase. In gene ontology phase, the gene ontology has been decomposed into three subontologies, the needed terms' attributes have been extracted from gene ontology and the subontologies have been normalized into one format. This phase has been applied when the system initiated. In annotation phase, the annotated index has been built from corpus documents using subontologies concepts and the system has been prepared for the searching process.

#### 3.1.1. Gene Ontology Phase

**Figure 2:**
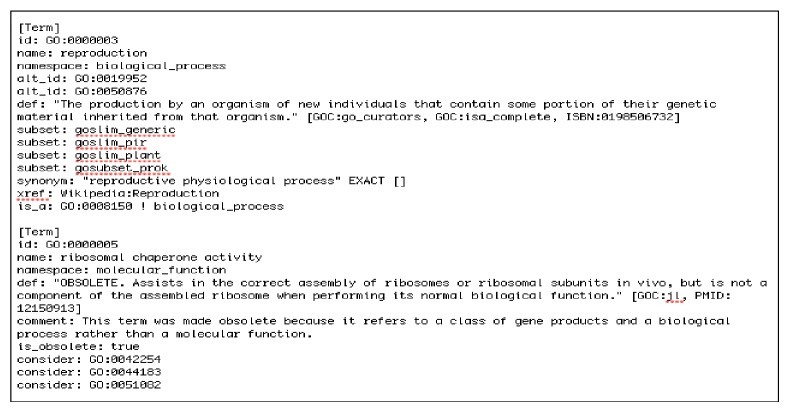
A sample of GO text file.

The gene ontology provides ontology of defined terms representing gene product properties [5]. The proposed system uses the gene ontology version 1.2, which is a text file that contains 37,486 gene terms.  Figure 2 shows a sample of GO. In this research the GO has been decomposed into three subontologies, normalized and stored in database to fulfill the proposed systems' objective of finding documents that contain function or process that can be applied on specific cellular component and make sure that the transaction will be smoother and faster. The GO decomposition has two main steps.

*(i) Extracting the Needed Attributes from Gene Ontology*. GO is stored as structured format. Each [Term] in the gene ontology contains different attributes that describe the term. Not all attributes in [Term] are needed to the proposed system. So the only needed attributes have to be extracted such as Term ID, term name, term namespace, term relations, term relation type, term synonyms, and synonyms type. To decompose GO into three subontologies and extract the needed terms' attributes, for each [Term] in the GO text file, the system checks for its namespace (type) and gets the needed attributes only then categorize them with one of three subontologies (cellular component, molecular function, and biological process).

*(ii) Storing the Needed Attributes in Normalized Database*. In this step, the data which has been extracted to represent the three subontologies has been stored in three different tables with the same format to make the transaction easier and faster. Each of the three subontology tables are connected with other two tables, which are synonyms and relations tables, which will be used later in the query.

#### 3.1.2. Annotation Phase

**Figure 3:**
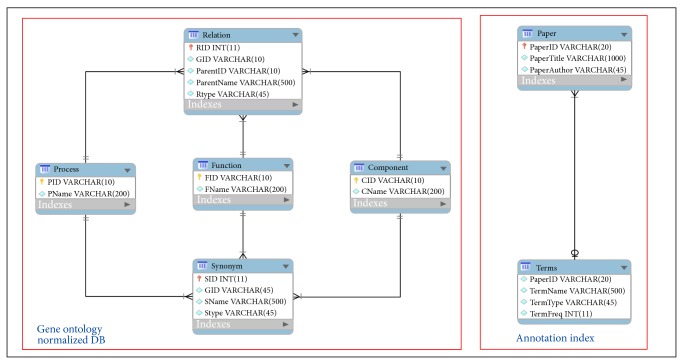
The normalized GO and the created annotation index.

Corpus, which consists of a lot of biological resources, has been annotated with the GO terms and the annotated index has been built which will be used in searching process. To annotate biological resources, for each document the system extracts the biological terms based on the normalized GO database. Special regular expressions have been used to annotate terms from biological documents. The used regular expressions are as follows.

(“∖∖s+” + term + “∖∖s+”) or (“∖∖/” + term + “∖∖s+”) or (“∖∖s+” + term + “∖∖.”) or (“∖∖.” + term + “//s+”) or (“∖∖^∧^” + term + “//s+”) or (term) where term is a concept that exists in the GO and is included in an annotated document.  Figure 3 shows the normalized GO and the created annotation index.  Table 1 describes the variables' meaning of normalized GO and the created annotation index in database design.

### 3.2. Search Level

**Figure 4:**
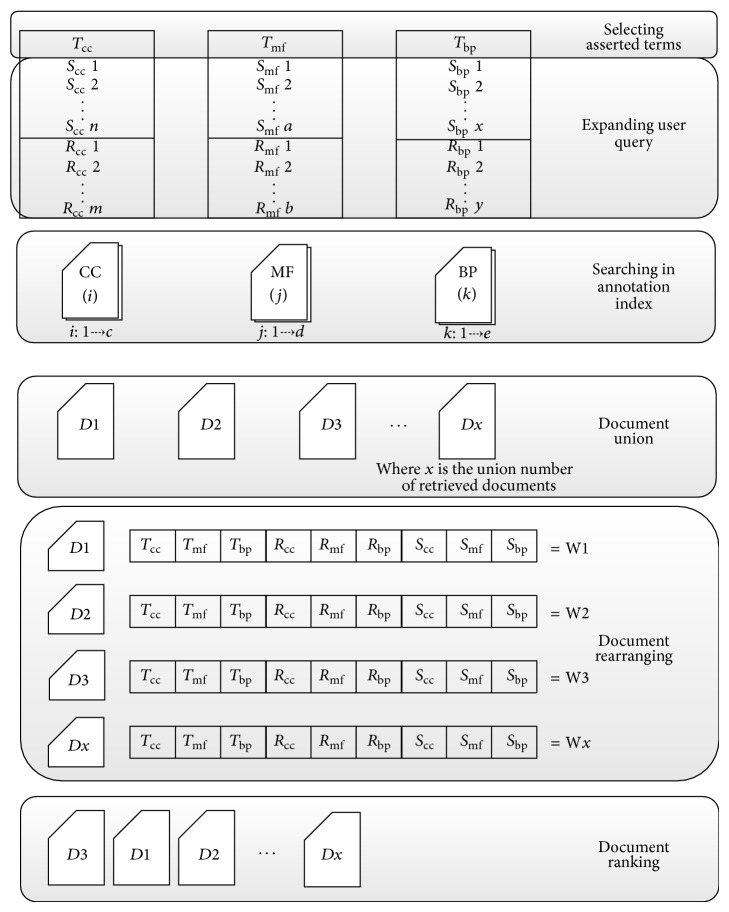
The different phases of search.

This is the second level of the system workflow. In the search level the user can select the searching terms from different subontologies to start the searching process. The system will expand the user terms by inferring other terms from normalized database that help semantically improve the result set. After retrieving the relevant documents the system will arrange and rank them to match the user priorities. The different phases of search are shown in Figure 4.

#### 3.2.1. Selecting Asserted Terms

The user can find and select searched terms from 3 different dropdown lists for 3 different subontologies terms. The user has the ability to choose the asserted terms and search for documents that either have cellular components, molecular functions, biological process, or any combinations of them.

#### 3.2.2. Expanding the User Query

The system infers the relevant terms to the asserted terms. For each asserted term, it extracts the term's synonyms and the term's relations from the normalized GO database. The inferred terms are used to improve the search results. For example, if the user wants to search for a biological process called “brain development” so the basic query will be* “select PaperID from terms where TermName = brain development.”* But when applying the query expansion, new queries will be created that contain the inferred term. The queries will be* “select PaperID from terms where TermName = organ development,” “select PaperID from terms where TermName = brain development,”* and* “select PaperID from terms where TermName = central nervous system development.”* The reason is that the “organ development” is a parent of the “brain development,” and “brain development” is part of the process called “central nervous system development.” So there is* “is_a”* relationship between the “brain development” and “organ development” and a* “part_of”* relationship between the “brain development” and “central nervous system development.” Each term from inferred terms will be in separated query.

#### 3.2.3. Searching in the Annotated Index

The system starts searching in the annotated index for documents containing any combination of terms (*T*), synonyms (*S*), and relations (*R*). Suppose that the three terms are cellular component term (*T*_cc_), molecular function term (*T*_mf_), and biological process term (*T*_bp_). The system will search for all synonyms and relations that related to each term and create list of synonyms with synonyms' type and relations with relations' type for each term. The synonyms' lists are cellular component terms' synonyms (*S*_cc_), molecular function terms' synonyms (*S*_mf_), and biological process terms' synonyms (*S*_bp_). Also list of relations which are cellular component terms' relations (*R*_cc_), molecular function terms' relations (*R*_mf_), and biological process terms' relations (*R*_bp_) will also be created. After searching, the system returns documents that contain terms, synonyms, and relations with the frequencies in each document. The result set is a number of documents that have these terms (i.e., documents per biological terms), but the search results are needed in another view; the view of documents per number of biological content, which are asserted terms and inferred terms or any combinations of them.

#### 3.2.4. Document Union

When the researcher searches for any combination of the three subontologies, there is a probability to get redundant documents. That is because the system retrieves documents based on just one term, synonym, or relation. So the search result may contain one document that retrieved twice for different terms. So, in this phase the system gets the union of all the documents to eliminate the redundant documents. After finishing this phase, the system will get only unique documents with unrepeated results.

#### 3.2.5. Document Rearranging

The system rearranges the documents using the following technique; for each document there is metadata structure that looks like this [*T*_cc_, *T*_mf_, *T*_bp_, *R*_cc_, *R*_mf_, *R*_bp_, *S*_cc_, *S*_mf_, *S*_bp_] where each entry in this list is the frequency of term in represented document with considering that each entry for synonym or relation is a list to represent different synonyms or relations found in the document.

#### 3.2.6. Document Ranking

The retrieved documents are ranked using special criteria; these criteria can be changed by the users of the system. As mentioned before, this system searches for documents using asserted terms in addition to inferred terms. These terms which are biological terms, terms' synonyms, and terms' relations have been used to calculate the ranking value of each retrieved document. The documents will be arranged according to the ranking values of these documents. Weights have been assigned for each of these terms. According to assigned weights each document can get ranked value using the following formula:

(1) DRank=WT(Tcc+Tmf+Tbp) +∑i=1m(Si∗Wsyn(i))+∑j=1nRj∗Wrel⁡j,

where *D*_Rank_ is the document ranking value and “*W*_*T*_” is the searched term's weight; it is the weight of any term type (cc, mf, or bp).

*T*_cc_, *T*_mf_, and *T*_bp_ are the frequencies of the cellular components, molecular function, and biological process terms in a document, respectively.

*W*_syn_(*i*) are the synonyms weights where *i* can take value from 1 to 4 to represent different types of synonyms in gene ontology which are four types; they will be assigned weight values as follows.

 
*W*_syn_(1) is the weight for “NARROW” synonyms defined in GO as “the term is narrower or more precise than the term name.”
 
*W*_syn_(2) is the weight for “EXACT” synonyms defined in GO as “an exact equivalent, interchange with the term name.”
 
*W*_syn_(3) is the weight for “RELATED” synonyms defined in GO as “terms are related in some way not covered above.”
 
*W*_syn_(4) is the weight for “BROAD” synonyms defined in GO as “the synonym is broader than the term name.”

*S*_*i*_ is the frequencies of these 4 different synonym types in the document.

*W*_*rel*⁡_(*j*) are the relations weights where *j* can take value from 1 to 4 to represent different types of relations in gene ontology which are four types; they will be assigned weight values as follows.

 
*W*_*rel*⁡_(1) is the weight for* “is_a”* relation.
 
*W*_*rel*⁡_(2) is the weight for* “has_part”* relation.
 
*W*_*rel*⁡_(3) is the weight for* “part_of”* relation.
 
*W*_*rel*⁡_(4) is the weight for* “regulates”* relation.

*R*_*j*_ is the frequencies of different types of relations in the document.

After calculating the documents' ranking values using the previous formula, the system rearranges the documents from higher to lower according to *D*_rank⁡_.

**Figure 5:**
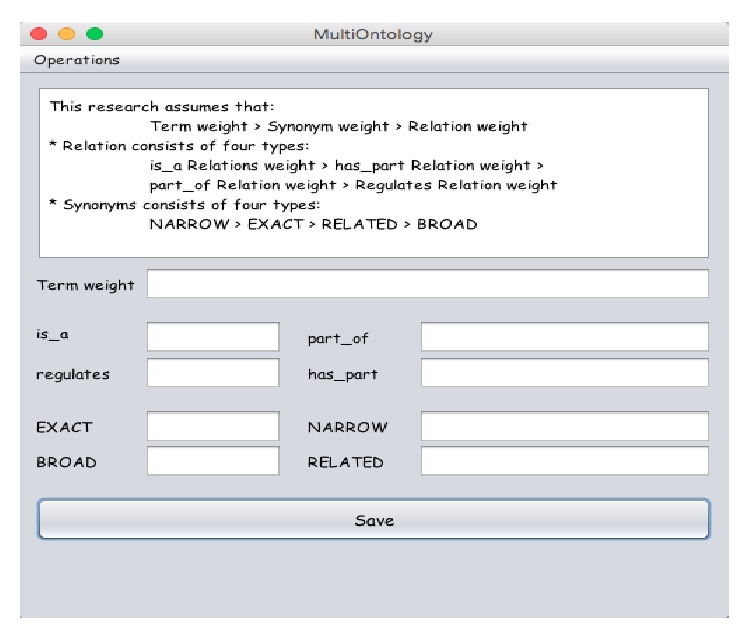
Snapshot from program interface for users' customized ranking criteria window.

The proposed system gives the users the flexibility in ranking documents according to his/her criteria by allowing him/her to enter customized weights. So the system has two options for ranking: either using the system default ranking values or using customized ranking based on the user customized values as shown in Figure 5.

The default values are assigned assuming that *W*_*rel*⁡_(1) > *W*_*rel*⁡_(2) > *W*_*rel*⁡_(3) > *W*_*rel*⁡_(4) for relations' weights and *W*_syn_(1) > *W*_syn_(2) > *W*_syn_(3) > *W*_syn_(4) for synonyms' weights. This research also assumes that *W*_*T*_ > *W*_Syn_ > *W*_Rel_. These assumptions suppose that the biological terms are exactly the researcher's needs, so they take the higher value. Synonyms get higher rank than relation because synonym is a representation of the term with difference in vocabulary or letters; it is useful in retrieving documents by authors who use different wording in reference to the same term. So it will get the second priority. Finally the relation will take the lowest weight because it is very generic to the term but it is also related to the term.

## 4. Illustrative Example

**Figure 6:**
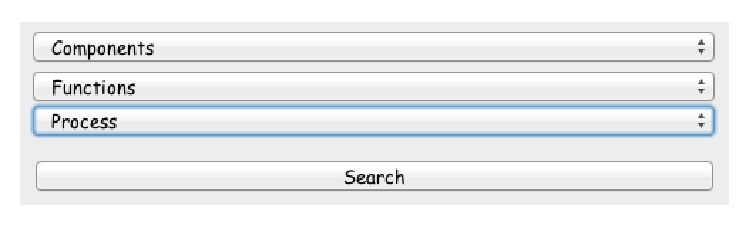
Snapshot from program interface for the three dropdown input lists.

When the researcher wants to search for the asserted terms in the proposed system, he/she can select three different terms from three different dropdown lists for* cellular components, molecular function,* and* biological process* as shown in Figure 6. So the researcher has the ability to search for

- one term (cc or mf or bp),
- two terms (any combination of the three components),
- three terms.

So when the terms have been chosen, the system starts gathering the inferred synonyms and relations that related to the chosen terms from the gene ontology. Then the query has been expanded using the inferred terms to add semantic value to the searching process. This example has been applied on “CRAFT” corpus [15] to clarify how the system works. Suppose that the user searches for a function called “binding” in “CRAFT” corpus; the system will create the metadata file for the searched term like the following:

 Term Name: binding
 ID: GO:0005488
 Type: Function
 Relations: molecular_function
 Synonyms: ligand NARROW

Then the system starts searching in the annotated index to match the metadata file with the annotated index fields and the result will be as shown in Table 2.

At the end of the searching the results will be a collection of related documents, and the system will rank these documents based on the frequency of the terms (cc, mf, bp, synonyms, and relations) in each document and the weight of each term. So the more ranked documents are the higher order in the list. And the final result of the documents names with the ranking value is shown in Table 3. Supposing that *W*_*T*_ = 10.0, *W*_Syn_(1) = 8.0, *W*_Syn_(2) = 7.0, *W*_Syn_(3) = 6.0, and *W*_Syn_(4) = 5.0, *W*_*rel*⁡_(1) = 4.0, *W*_*rel*⁡_(2) = 3.0, *W*_*rel*⁡_(3) = 2.0, and *W*_*rel*⁡_(4) = 1.0.

## 5. Experimental Results

A set of experiments are performed to study the efficiency and effectiveness of the proposed system. All test cases are tested using gene ontology version 1.2 and CRAFT corpus version 1.0.

**Figure 7:**
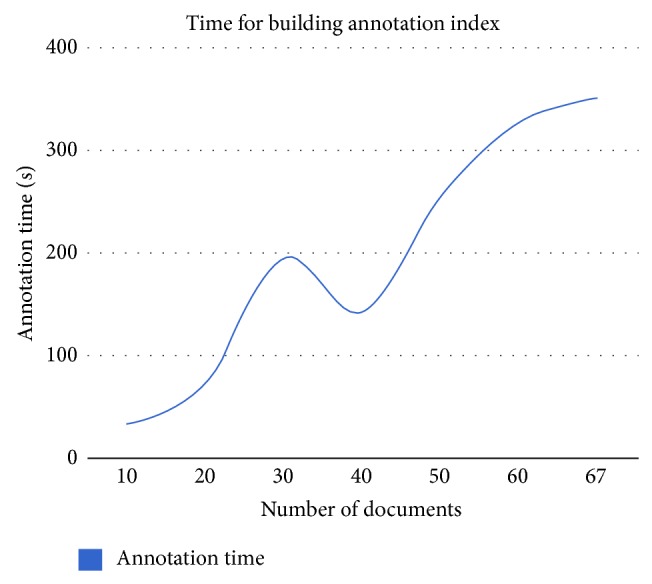
Annotation time versus number of documents.

The system tries to improve the searching process using annotation technique. Annotation technique extracts the biological terms involved in the gene ontology from documents.  Table 4 and Figure 7 show the relation between number of documents and annotation time in seconds to build annotated index for CRAFT corpus.

**Figure 8:**
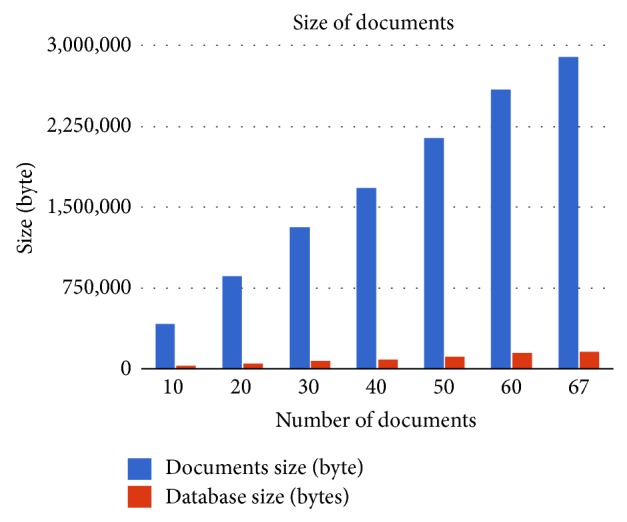
Comparison between documents' sizes and annotated index size.

Using annotation, the search process will be done in annotated index instead of searching in the whole documents. Since the size of annotated index is smaller than the corpus size, the search process will be faster.  Table 5 and Figure 8 show a comparison between documents size in bytes before annotation and the size of annotated index for the corpus.

**Figure 9:**
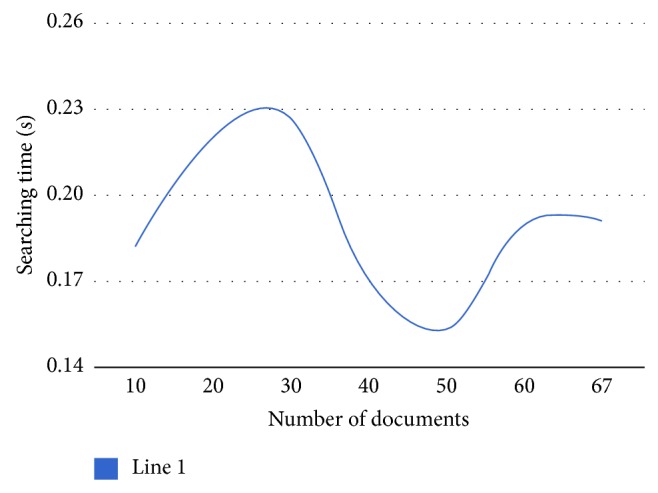
Searching time via number of documents.

One of the most important aspects in evaluating retrieval systems is searching time.  Table 6 and Figure 9 show the relation between the average times of searching in the annotated index in seconds with the increasing number of documents used in building the annotated index.

*Test Case 1*. In this test case the system will take a cellular component term only as an input to verify the one term search process as shown in Tables 7 and 8.

*Test Case 2*. In this test case the system will take a cellular component term and a function term as an input to verify the two terms search process as shown in Tables 9 and 10.

*Test Case 3*. In this test case the system will take a cellular component term, a molecular function term, and a biological process term as an input to verify the three terms search process as shown in Tables 11 and 12.

## 6. Conclusion and Future Work

We have developed ontology-based information retrieval system that retrieves relevant documents to the searching inputs with high performance using annotated index. The system decomposes the GO semantically into three subontologies. The system retrieves related documents by focusing only on extracted biological terms based on GO and building annotated index using these terms. The system has improved the performance of information retrieving method, since it has used the biological ontology to get the synonymous and the relations of the asserted terms in the query to retrieve all relevant documents that contain the terms and their synonyms and relations. The system has used ranking criteria to rank the retrieved documents. Also researchers have the flexibility to include their ranking values to help them in ordering retrieved documents according to their criteria. This system has been tested using GO version 1.2 and CRAFT version 1.0.

## Figures and Tables

**Table 1:** The normalized GO and the created index description.

	Table name	Variable name	Variable description
Gene ontology normalized database	Component	CID	Cellular component Term ID
		CName	Cellular component name
	Function	FID	Molecular function Term ID
		FName	Molecular function name
	Process	PID	Biological process Term ID
		PName	Biological process name
	Relation	RID	Relation ID (auto number)
		GID	Gene ID (Term ID in GO)
		ParentID	Parent Term ID
		ParentName	Parent term name
		RType	Relation type (is_a, part_of, has_part, regulates)
	Synonym	SID	Synonym ID (auto number)
		GID	Gene ID (Term ID in GO)
		SName	Synonym name
		SType	Synonym type (EXACT, NARROW, BROAD, RELATD)

Annotated index	Paper	PaperID	Paper ID (paper name)
		PaperTitle	Paper title
		PaperAuthor	Paper author
	Term	PaperID	Paper ID (paper name)
		TermName	Term name
		TermType	Term type
		TermFreq	Term frequency

**Table 2:** Result of term existence in documents based on cc, mf, and bp.

	cc	mf	bp
Terms	0	42	0
Relations	0	0	0
Synonyms	0	10	0
Number of found documents (union)	45 documents		

**Table 3:** List of arranged documents based on the ranking criteria.

Document name	Document ranking
PUBMED (10).txt	340
PUBMED (1).txt	320
PUBMED (58).txt	270
PUBMED (19).txt	170
PUBMED (35).txt	140
PUBMED (43).txt	110
PUBMED (4).txt	90
PUBMED (36).txt	80
PUBMED (55).txt	80
PUBMED (56).txt	80

**Table 4:** Annotation time based on the number of documents.

Number of documents	Annotation time (seconds)
10	33.698
20	72.859
30	195.381
40	143.111
50	256.965
60	327.658
67	351.658

**Table 5:** Comparison between documents' sizes and annotated index size.

Number of documents	Document size (bytes)	Annotated index size (bytes)
10	412,161	21,510
20	869,791	44,393
30	1,316,576	69,676
40	1,686,597	90,165
50	2,145,198	113,120
60	2,592,895	140,526
67	2,908,446	156,294

**Table 6:** Searching time via the number of documents.

Number of documents	Searching time in second
10	0.182
20	0.220
30	0.227
40	0.171
50	0.153
60	0.190
67	0.191

**Table 7:** Extracted terms.

Cellular component term	Chromosome
Synonyms	Chromatid (related) Interphase chromosome (narrow) Prophase chromosome (narrow)

Relations	Intracellular non-membrane-bounded organelle (is_a)

**Table 8:** Count of relevant documents.

	Cellular component
Terms	31
Relations	0
Synonyms	2

Number of relevant documents	

31	

**(a):** 

Cellular component term	Nucleus

Synonyms	Cell nucleus (exact)

Relations	Intracellular non-membrane-bounded organelle (is_a)

**(b):** 

Function term	Alkaline phosphatase activity
Synonyms	Alkaline phenyl phosphatase activity (exact) alkaline phosphohydrolase activity (exact) Alkaline phosphomonoesterase activity (exact) glycerophosphatase activity (broad) orthophosphoric-monoester phosphohydrolase (alkaline optimum) (exact) Phosphate-monoester phosphohydrolase (alkaline optimum) (exact) phosphomonoesterase activity (broad)

Relations	Phosphatase activity (is_a)

**Table 10:** Count of relevant documents.

	Component	Function
Terms	30	1
Relations	0	2
Synonyms	5	0

Number of relevant documents		

30		

**(a):** 

Cellular component term	Host

Synonyms	Host organism (exact)

Relations	Other organisms (is_a)

**(b):** 

Function term	Binding

Synonyms	Ligand (narrow)

Relations	Molecular_function (is_a)

**(c):** 

Process term	Growth

Synonyms	Growth pattern (related) Nondevelopmental growth (related)

Relations	Biological_process (is_a)

**Table 12:** Count of relevant documents.

	Component	Function	Process
Terms	5	42	36
Relations	3	0	0
Synonyms	0	10	2

Number of relevant documents			

56			
